# Genomic SEM reveals shared genetic architecture and expands locus discovery for locomotor activity in heterogeneous stock rats

**DOI:** 10.64898/2026.10.01.756043

**Published:** 2026-10-06

**Authors:** Apurva S. Chitre, Thiago Missfeldt Sanches, Gavrila Ang, Nana K. Amissah, Rodolfo Avila, Hannah Bimschleger, Paola Campo, Nazzareno Cannella, Denghui Chen, Riyan Cheng, Katarina A. Cohen, Ayteria D. Crow, Eric Dereschewitz, Michelle R. Doyle, Maya Eid, Anthony M. George, Aidan P. Horvath, Keita Ishiwari, Benjamin B. Johnson, Elaine Keung, Christopher King, Connor D. Martin, Angel Garcia Martinez, Daniel Munro, Alesa H. Netzley, Khai-Minh Nguyen, Beverly Peng, Dominika Pullmann, Sara R. M. U. Rahman, Analyse T. Roberts, Mohammad Sadegi, Deborah Sevigny-Resetco, Laura Soverchia, Celine L. St. Pierre, Nina Suzuki, Brady M. Thompson, Shambhavi Tyagi, Massimo Ubaldi, Robert M. Vogel, Tengfei Wang, Yizhi Wang, Hao Chen, Roberto Ciccocioppo, Giordano de Guglielmo, David Dietz, Shelly B. Flagel, Thomas Jhou, Jicai Jiang, Peter W. Kalivas, Brittany N. Kuhn, Paul J. Meyer, Suzanne H. Mitchell, Terry E. Robinson, Oksana Polesskaya, Abraham A. Palmer

## Abstract

Locomotor response to a novel environment is a heritable, addiction-relevant behavioral phenotype in rodents. We characterized its genetic basis in 9,341 male and female heterogeneous stock (HS) rats from eight studies. Locomotor activity was heritable across studies, while pairwise genetic correlations were positive but variable, indicating shared yet nonidentical genetic influences. Given nonuniform genetic architecture, we compared three complementary approaches to locus discovery that analyzed SNP effects 1) on a pooled locomotor phenotype (MEGA), 2) across studies under a fixed-effect model (METAL), or 3) on a shared genetic factor (Genomic SEM). To ensure comparability, all three locus-discovery approaches were applied to a common subset of seven studies. Together, these approaches identified 38 loci, with both convergence and method-specific discovery across analyses. Genomic SEM identified 31 loci, compared to 8 for MEGA and 13 for METAL. To interpret the loci, we integrated brain molecular QTLs, predicted coding and splice consequences, and open-field locomotor phenotypes from knockouts of orthologous mouse genes. Our results highlighted established neurobehavioral candidates spanning locomotor and reward-related biology ( *Negr1* and *Lmo4* ), serotonergic signaling ( *Htr1b* ), and GABAergic signaling ( *Gabrg2* ), as well as relatively novel genes including *Tcerg1l, Pcdh10,* and *Tmem132e* . Our results illustrate how Genomic SEM can integrate genetically correlated yet heterogeneous behavioral measures and enable discovery of loci underlying their shared genetic architecture, highlighting its utility for complex-trait studies in rodents.

## Full Text Availability

The license terms selected by the author(s) for this preprint version do not permit archiving in PMC. The full text is available from the preprint server.

